# Facelift Surgery and Nerve Injury: A Systematic Review and Meta-Analysis

**DOI:** 10.1007/s00266-025-04932-7

**Published:** 2025-06-02

**Authors:** Gonçalo Gandra, Bia S. Silva, Ricardo Horta

**Affiliations:** 1 Department of Plastic, Reconstructive and Aesthethic Surgery, and Burn Unity, Centro Hospitalar de São João, Porto, Portugal; 2 Department of Surgery and Physiology, Faculty of Medicine, University of Porto, Porto, Portugal; 3 Faculty of Medicine, University of Porto, Porto, Portugal; 4 Gondomar, Portugal

**Keywords:** Facelift, Facial nerve, Nerve injury

## Abstract

**Background:**

Advances in surgical procedures improved the safety profile of aesthetic surgery. Several side effects have been described for facelift surgeries, and nerve injuries are one of the most feared due to their impact on quality of life. The main objective of this work is to evaluate the rate and type of nerve injury during facelift procedures through a systematic review.

**Methods:**

PubMed®, EMBASE® and Web of Science® databases were searched for articles on nerve injury rates after facelift surgeries using controlled and non-controlled terms to establish search queries. Three investigators independently assessed the eligibility of publications, first based on the title and abstract and then based on the full text. The DerSimonian-Laird random effects model was used for proportion estimation through a meta-analysis.

**Results:**

A total of 67 eligible publications with a total of 15,404 patients and 15,441 procedures were included in the analysis according to the eligibility criteria. The overall estimated pooled motor and sensory nerve damage rate was 0.66% (95% confidence interval [95%CI]: [0.5%; 0.9%], Z = 6.07, p < .001) and 0.39% (95%CI: [0.2%; 0.6%], Z = 4.16, p < .001), respectively. For permanent neuronal damage, the estimated pooled rates were 0.047% (95%CI: [0.0%; 0.1%], Z = 2.69, *p* = .007) and 0.045% (95%CI: [0.0%; 0.1%], Z = 2.63, *p* =.009), respectively, for motor and sensory nerve damage.

**Conclusions:**

The recognition of nerve damage as a serious complication of facelift surgery is increasing, although the estimated pooled rate is less than 1%.

**Level of Evidence II:**

This journal requires that authors assign a level of evidence to each article. For a full description of these Evidence-Based Medicine ratings, please refer to the Table of Contents or the online Instructions to Authors  www.springer.com/00266.

## Introduction

In recent decades, advances in surgical methods have allowed aesthetic surgery to be performed with greater safety, perhaps in response to an increasing demand for aesthetic procedures [1]. One of the possible reasons for this increased demand is the increase in the elderly population, more affected by tissue deflation and ligament laxity, ultimately reflecting the facial aspect [2]. In 2020, the American Society for Aesthetic Plastic Surgery reported 188 193 aesthetic procedures for the face, neck, and brow, with 59906 facelifts, midface lifts, or combination of facelifts [3].

A facelift, also known as rhydectomy, "is a surgical procedure that aims to rejuvenate facial soft tissues to achieve a younger and harmonious appearance," according to Yang and Hohman [4]. A variety of nonsurgical methods, including pulsated light, radiation therapy, and acid peels, can be used to rejuvenate the face.

However, surgical methods have been shown to be more effective than these treatments alone, resulting in better and more durable results. For this reason, facelift surgery is considered the gold standard for facial rejuvenation. To achieve synergistic aesthetic results, surgical and nonsurgical techniques have recently merged. In particular, some experts support a multimodal approach to contemporary facial rejuvenation that includes soft tissue fillers, neuromodulators, and surgical procedures [5]. The desired results, the anatomical characteristics of the patient, and the evaluation of potential problems all play a role in selecting the technique that best fits a given situation [6, 7].

The initial procedure for a traditional facelift consisted of manipulation of the subcutaneous flap layer, which later evolved to a procedure that incorporates deeper areas of the fascial layer to achieve better and longer results. Facelift techniques that encompass deeper facial layers resulted from a better understanding of the superficial musculoaponeurotic system (SMAS), first described in 1976 [8]. Current surgical facelift approaches are based on SMAS tightening to ensure elevation of facial soft tissues in a vertical plane, with the goal of fading wrinkles and skin sagging. Procedures that address the lower face and neck through SMAS tightening can be classified as low-SMAS interventions. On the other hand, extended SMAS procedures address the midface, including the nasolabial fold, by manipulating the SMAS and the zygomatic and masseteric ligaments. Low-SMAS facelifts include SMASplication, where the SMAS is tightened by pulling [9], SMAS imbrication, where the SMAS is tightened while folded and sutured [10], and SMASectomy, where tightening is achieved by excision of a small portion of the SMAS, with reattachment of the remaining part of the SMAS. Extended SMAS facelifts include deep-plane rhytidectomy, which is considered the gold standard technique, where zygomatic and masseteric ligaments are released to further pull the SMAS [7, 11], high SMAS procedures (including variations), where the SMAS is elevated upward through different techniques [12, 13], and composite rhytidectomy, corresponding to the combination of different types of facelift [11, 14].

Most facelift surgeries require general anesthesia, allowing precise facial dissection [11]. Postoperative complications have been reported for facelift procedures, including swelling, bruising, scarring, discomfort, hematomas, skin necrosis, infection, keloid scarring, and nerve injury, among others [15]. Nerve injury is a major problem aesthetic surgeons should consider during facelift procedures, especially damage to the facial nerve. In fact, although more aggressive techniques guarantee better and more lasting results, they also increase the risk of nerve damage. Selection must include the assessment of the different risks and benefits so that the patient to be fully aware of the decision [16].

Although deeper dissections occur closer to the nerve branches, there are still many reports of nerve injury in subcutaneous dissections [17]. A better understanding of the distribution within the face is the key to minimizing their damage during facelift procedures. Furthermore, other aesthetic procedures, such as liposuction, can induce nerve damage, and their combination with facelift surgery does not necessarily prevent or minimize the rate of post-procedure nerve lesions [18].

Taking into account the burden of nerve injury on patient quality of life and the heterogeneity of reports on this surgical facelift complication, the main objective of this work was to evaluate the rate and type of nerve injury in patients undergoing facelift procedures, through a systematic review of the literature.

## Materials and Methods

### Literature Search and Study Selection

A systematic review of the literature was performed using the Preferred Reporting Items for Systematic Reviews and Meta-Analyses (PRISMA) guidelines. The literature search was performed in PubMed®, EMBASE® and Web of Science® databases using a combination of controlled terms (MeSH and EMTREE) and synonyms, last confirmed in January 2022. The following MeSH terms were used: “Rhytidoplasty” [Mesh], “Cranial Nerve Injuries”[Mesh], “Wounds and Injuries”[Mesh]; the correspondent EMTREE terms were as follows: ‘rhytidoplasty’, ‘nerve’, ‘injury’, ‘wound’, ‘lesion’. The literature search was carried out independently by three investigators without restriction on time or sample size.

The resulting search queries according to each database were, for PubMed® (“Rhytidoplasty”[Mesh] OR Rhytidoplasty OR facelift OR “face lift” OR “face lifts” OR Platysmotomy OR Rhytidectomy OR Platysmaplasty) AND (“Cranial Nerve Injuries”[Mesh] OR “nerve injury” OR “nerve injuries” OR ((“Cranial Nerves”[Mesh] OR “cranial nerve” OR “nerve”) AND (“Wounds and Injuries”[Mesh] OR trauma* OR injur* OR wound* OR lesion* OR damag*))); for EMBASE® (‘rhytidoplasty’/exp OR rhytidoplasty OR ‘facelift’ OR ‘face lift’ OR ‘face lifts’ OR platysmotomy OR rhytidectomy OR platysmaplasty) AND (‘nerve’/exp OR ‘nerve’) AND (‘injury’/exp OR ‘injury’ OR injur* OR ‘wound’/exp OR ‘wound’ OR wound* OR trauma* OR ‘lesion’/exp OR ‘lesion’ OR lesion* OR damag*); and for Web of Science® ((nerve OR nerves) AND (injur* OR damag* OR wound* OR trauma*)) AND (Rhytidoplasty OR facelift OR “face lift” OR “face lifts” OR Platysmotomy OR Rhytidectomy OR Platysmaplasty).

The following inclusion criteria were considered: (1) patients over 18 years old; (2) patients who underwent facelift surgery; (3) description of the rate of nerve damage; (4) description of the type of nerve damage; and (5) observational or experimental studies such as case series or clinical trials. Studies were excluded if: (1) they were written in other languages than English, Portuguese, Spanish or French; (2) duplicate data; (3) other study design (case reports, comments, series, reviews, and editorials); (4) description of surgery for non-aesthetic purposes (such as the treatment of mandible fractures, parotidectomy, or thyroidectomy); (5) cadaver dissections for anatomic description; and (6) not available or insufficient data.

### Data Extraction

According to the PRISMA guidelines, each step was carried out independently by two investigators. Briefly, manuscripts were first selected by analyzing titles and abstracts based on inclusion/exclusion criteria. The full texts were then reviewed and data extracted (first author, year of publication, original country, number of patients and surgical interventions, age, gender, type of facelift surgery, adjunctive procedures, overall nerve damage, and permanent nerve damage rates). Studies were evaluated for the potential risk of bias, namely, confounding, selection, information, and reporting bias. The risk of confounding bias was evaluated through the presence of other potential causes of nerve damage for which the outcome was not specifically reported; Selection bias risk was considered whenever a specific sample was included in the study that could compromise the frequency of nerve damage; risk was considered when the type of nerve injury or type of surgery was not clearly reported to quantify our outcome; Report bias was considered whenever the result was only reported for a specific group of patients.

### Statistical Analysis

Meta-analysis was performed with open source software Jamovi, version 1.6.23, using the METAFOR package [19–21]. The pooled proportion of the rate of nerve damage was estimated according to the DerSimonian-Laird random effects model and the heterogeneity between the studies was assessed using the Cochrane Q test and determination of I^2^. A *p*-value less than 0.05 was considered statistically significant.

## Results

### Study Selection and Inclusion

**Fig. 1:**
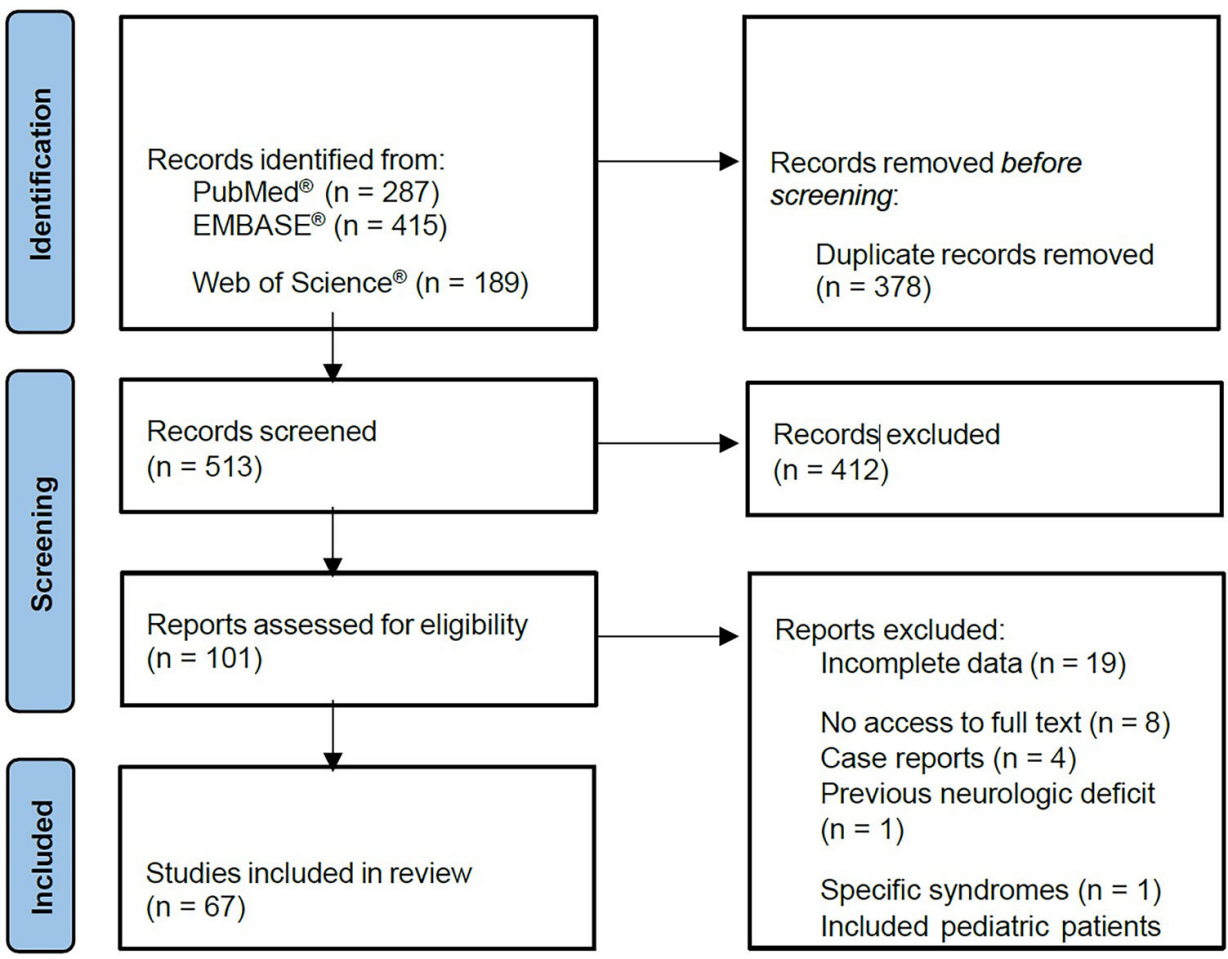
PRISMA flow chart for systematic review and meta-analysis

The study selection flow diagram is presented in Figure 1. PubMed® provided a total of 287 manuscripts, while EMBASE® showed 415 results, and Web of Science® provided a total of 189 publications. After duplicate removal, a total of 513 records were screened by title and abstract, with a total of 412 articles excluded due to the following reasons: publication in languages not included in the inclusion criteria, anatomy studies in cadavers, description of surgical techniques, letter to the editor, no assessment/report on neurological damage or type of surgery, case reports, review articles and meta-analysis. A total of 101 manuscripts were evaluated for complete review, with the exclusion of 34 studies due to incomplete data (n = 19), no access to full text (n = 8), case reports (n = 4), treatment of patients with previous neurologic deficits (n=1), specific syndromes (n = 1) and included pediatric patients (n=1). The analysis resulted in the inclusion of a total of 67 publications [6, 22–87]. Among the included studies, we observed that 38 were carried out in the United States of America, 10 in Europe, 7 in Asia, 6 in South America, 4 in more than one country, and the remaining in South Africa and Australia.

### Study and Patient Characteristics

Table 1 summarizes the characteristics of the 67 included publications (43 retrospective series, 12 prospective series, and 12 studies with missing information), comprising a total of 15 404 patients and 15 441 procedures, with studies ranging from 8 to 3 580 participants. Taking into account the publications reporting gender and age, all except one [79] included more female patients, ranging from 42.9 to 100%, and the age ranged from 21 to 88 years.

**Table 1:** Characteristics of the studies included in the systematic review

Authors, year	Country	Patients/Procedures	Age (years), Mean (range)	Females (%)	Type of Surgery	First face lift	Adjunctive Procedures	Motor Neural Lesion, n (%) Overall/Permanent	Sensory Neural Lesion,n (%) Overall/Permanent
Badin et al. 2001 [22]	Brazil	135/135	(35–55)	128 (94.8%)	Midface lift	N/R	N/R	5 (3.7%)/0 (0%)	0 (0%)/0 (0%)
Bass 1998 [23]	USA	20/20	53±7.5(45–73)	18 (90%)	Midface lift	18 (90%)	N/R	0 (0%)/0 (0%)	0 (0%)/0 (0%)
Baylis et al. 2000 [24]	USA	395/395	N/R	N/R	Midface lift	N/R	N/R	2 (0.5%)/1 (0.3%)	0 (0%)/0 (0%)
Beale et al. 2013 [25]	USA	60/60	60.5 (46–77)	57 (95%)	Midface lift	0 (0%)	N/R	1 (1.7%)/0 (0%)	0 (0%)/0 (0%)
Berry et al. 2010 [26]	UK	117/117	55 (29–79)	109 (93.2%)	Midface lift	88 (75.2%)	Overall: 104 (88.9%) Blepharopl.: 55 (47%) Browpexy: 26 (22.2%) Rhynoplasty: 14 (12%)	4 (3.4%)/0 (0%)	1 (0.9%)/1 (0.9%)
Castello et al. 2011 [27]	Italy	327/327	51.1 (38–75)	316 (96.6%)	Midface lift	N/R	Overall: 178 (54.4%) Blepharopl.: 170 (52%)	2 (0.6%)/0 (0%)	0 (0%)/0 (0%)
Chisholm et al. 1995 [28]	USA	50/50	52.2 (41–68)	44 (88%)	Midface lift	N/R	Blepharopl.: 44 (88%) Browpexy: 17 (34%) Rhynoplasty: 30 (60%)	1 (2%)/0 (0%)	6 (12%)/0 (0%)
Choi et al. 2010 [29]	South Korea	10/10	23.3 ± 4.4	8 (80%)	Midface lift	10 (100%)	Overall: 10 (100%)	0 (0%)/0 (0%)	0 (0%)/0 (0%)
Choi et al. 2020 [30]	South Korea	179/179	44.4 ± 14.4	173 (96.6%)	Midface lift	N/R	N/R	0 (0%)/0 (0%)	0 (0%)/0 (0%)
Citarella et al. 2010 [31]	Brazil	54/54	38 (28–55)	35 (64.8%)	Midface lift	N/R	N/R	0 (0%)/0 (0%)	0 (0%)/0 (0%)
Colombo et al. 2015 [32]	Italy	31/31	(35–64)	25 (80.6%)	Midface lift	N/R	Blepharopl.: 31 (100%)	0 (0%)/0 (0%)	0 (0%)/0 (0%)
Cornette de Saint-Cyr et al. 2007 [33]	France	150/150	57 (31–88)	128 (85.3%)	Midface lift	N/R	Blepharopl.: 150 (100%)	1 (0.7%)/0 (0%)	37 (24.7%)/0 (0%)
De Cordier et al. 2002 [34]	USA	458/472	55.7 (31–88)	426 (93%)	Midface lift	458 (97%)	Blepharopl.: 243 (51.5%)	16 (3.4%)/0 (0%)	0 (0%)/0 (0%)
Delaney et al. 2019 [35]	USA	48/48	56.4 (28–73)	37 (77.1%)	Midface lift	39 (81.3%)	N/R	1 (2.1%)/0 (0%)	0 (0%)/0 (0%)
Duminy et al. 1997 [36]	South Africa	35/35	45.6 (32–69)	33 (94.3%)	Midface lift	N/R	Overall: 0 (0%)	0 (0%)/0 (0%)	0 (0%)/0 (0%)
Eremia et al. 2002 [37]	USA	60/60	58 (45–85)	60 (100%)	Midface lift	60 (100%)	Overall: 42 (70%) Belpharopl.: 21 (35%) Browpexy: 13 (21.7%)	1 (1.7%)/0 (0%)	0 (0%)/0 (0%)
Firmin et al. 2009 [38]	Australia	420/420	N/R	N/R	Midface lift	N/R	N/R	4 (1%)/0 (0%)	0 (0%)/0 (0%)
Foerster 1982 [6]	USA	266/266	53	258 (97%)	Midface lift	N/R	Blepharopl.: 164 (61.7%) Browpexy: 0 (0%) Rhynoplasty: 3 (1.1%)	0 (0%)/0 (0%)	0 (0%)/0 (0%)
Graf et al. 2003 [39]	Brazil	96/96	N/R	91 (94.8%)	Midface lift	N/R	Overall: 24 (25%)	0 (0%)/0 (0%)	0 (0%)/0 (0%)
Griffin et al. 2007 [40]	USA	178/178	58	156 (87.6%)	Midface lift	N/R	N/R	0 (0%)/0 (0%)	0 (0%)/0 (0%)
Guyuron 1988 [41]	USA	286/286	N/R	N/R	Midface lift	N/R	N/R	0 (0%)/0 (0%)	0 (0%)/0 (0%)
Guyuron et al. 2018 [42]	USA	72/72	58	64 (88.9%)	Midface lift	72 (100%)	N/R	0 (0%)/0 (0%)	0 (0%)/0 (0%)
Hagerty et al. 1998 [43]	USA	89/89	N/R	82 (92.1%)	Midface lift	72 (80.9%)	N/R	5 (5.6%)/0 (0%)	0 (0%)/0 (0%)
Heinrichs et al. 1998 [44]	USA	200/200	54±11 (34–76)	196 (98%)	Midface lift	188 (94%)	N/R	2 (1%)/0 (0%)	1 (0.5%)/0 (0%)
Hopping et al. 2010 [45]	USA	300/300	N/R	253 (84.3%)	Midface lift	N/R	N/R	4 (1.3%)/0 (0%)	0 (0%)/0 (0%)
Jacono et al. 2011 [46]	USA	153/153	57.8 (36–75)	146 (95.4%)	Midface lift	N/R	N/R	2 (1.3%)/0 (0%)	0 (0%)/0 (0%)
Kaye et al. 2016 [47]	USA	159/159	N/R	152 (95.6%)	Midface lift	N/R	Blepharopl.: 147 (92.5%) Browpexy: 24 (15.1%)	2 (1.3%)/0 (0%)	0 (0%)/0 (0%)
Keller et al. 1996 [48]	USA	73/73	N/R	61 (83.6%)	Midface lift	N/R	N/R	1 (1.4%)/0 (0%)	0 (0%)/0 (0%)
Le Louarn 2018 [49]	France	342/342	55.3 (21–89)	232 (67.8%)	Midface lift	N/R	N/R	0 (0%)/0 (0%)	2 (0.6%)/0 (0%)
Lee et al. 1998 [50]	USA	29/29	54.7 (40–77)	25 (86.2%)	Midface lift	N/R	N/R	0 (0%)/0 (0%)	0 (0%)/0 (0%)
Lemmon et al. 1980 [51]	USA	577/577	54 (35–87)	545 (94.5%)	Midface lift	567 (98.3%)	Overall: 552 (95.7%) Blepharopl.: 540 (93.6%) Browpexy: 18 (3.1%) Rhynoplasty: 19 (3.3%)	10 (1.7%)/0 (0%)	0 (0%)/0 (0%)
Lindsey 2009 [52]	USA	60/60	N/R	N/R	Midface lift	N/R	N/R	0 (0%)/0 (0%)	0 (0%)/0 (0%)
Liu et al. 2019 [53]	China	179/179	47.17	177 (98.9%)	Midface lift	N/R	Blepharopl.: 179 (100%)	0 (0%)/0 (0%)	0 (0%)/0 (0%)
McKinney et al. 1984 [54]	USA	77/77	N/R	N/R	Midface lift	N/R	Blepharopl.: 50 (64.9%)	2 (2.6%)/0 (0%)	0 (0%)/0 (0%)
Noone 2006 [55]	USA	259/259	N/R	N/R	Midface lift	N/R	Overall: 240 (92.7%)	0 (0%)/0 (0%)	0 (0%)/0 (0%)
Obourn et al. 2018 [56]	USA	95/118	N/R	N/R	Midface lift	N/R	N/R	0 (0%)/0 (0%)	0 (0%)/0 (0%)
Pascali et al. 2017 [57]	Italy	199/199	51.8 (29–78)	108 (54.3%)	Midface lift	N/R	N/R	0 (0%)/0 (0%)	1 (0.5%)/0 (0%)
Patrocínio et al. 2002 [58]	Brazil	62/62	48 (38–63)	60 (96.8%)	Midface lift	N/R	Overall: 0 (0%)	0 (0%)/0 (0%)	1 (1.6%)/0 (0%)
Pina 1997 [59]	Brazil	145/145	(40–74)	N/R	Midface lift	133 (91.7%)	Blepharopl.: 145 (100%)	0 (0%)/0 (0%)	4 (2.8%)/0 (0%)
Rammos et al. 2015 [60]	USA	229/229	61 (25–75)	202 (88.2%)	Midface lift	175 (76.4%)	Overall: 183 (79.9%) Blepharopl.: 85 (37.1%) Browpexy: 72 (31.4%)	9 (3.9%)/0 (0%)	0 (0%)/0 (0%)
Robbins et al. 1995 [61]	USA	226/226	(26–85)	208 (92%)	Midface lift	N/R	N/R	0 (0%)/0 (0%)	0 (0%)/0 (0%)
Ryu et al. 2015 [62]	Korea	53/53	50.7 (35–66)	47 (88.7%)	Midface lift	N/R	Blepharopl.: 13 (24.5%) Browpexy: 11 (20.8%)	1 (1.9%)/1 (1.9%)	0 (0%)/0 (0%)
Sadati et al. 2019 [63]	USA	110/110	62.2	107 (97.3%)	Midface lift	90 (81.8%)	Blepharopl.: 33 (30%) Browpexy: 5 (4.5%)	1 (0.9%)/0 (0%)	0 (0%)/0 (0%)
Serra-Renom et al. 2008 [64]	Spain	36/36	54 (42–74)	31 (86.1%)	Midface lift	N/R	N/R	0 (0%)/0 (0%)	0 (0%)/0 (0%)
Shauly et al. 2021 [65]	USA	241/241	61±8.2 (40–85)	212 (88%)	Midface lift	160 (66.4%)	Overall: 124 (51.5%)	0 (0%)/0 (0%)	0 (0%)/0 (0%)
Sullivan et al. 1999 [66]	USA	96/96	57 (45–73)	78 (81.3%)	Midface lift	N/R	Overall: 96 (100%)	3 (3.1%)/0 (0%)	6 (6.3%)/1 (1%)
Swanson 2020 [67]	USA	225/225	59.1 (41–79)	197 (87.6%)	Midface lift	N/R	Overall: 134 (59.6%)	17 (7.6%)/0 (0%)	0 (0%)/0 (0%)
Tanna et al. 2008 [68]	USA	1000/1000	57 (39–85)	950 (95%)	Midface lift	785 (78.5%)	Blepharopl.: 449 (44.9%)	0 (0%)/0 (0%)	0 (0%)/0 (0%)
Tellioglu et al. 2006 [69]	Turkey and Japan	16/16	N/R	16 (100%)	Midface lift	N/R	N/R	0 (0%)/0 (0%)	0 (0%)/0 (0%)
Thompson et al. 1978 [70]	USA	922/922	54 (29–80)	876 (95%)	Midface lift	N/R	Blepharopl.: 813 (88.2%) Browpexy: 2 (0.2%) Rhynoplasty: 155 (16.8%)	6 (0.7%)/1 (0.1%)	0 (0%)/0 (0%)
Ullmann et al. 2004 [71]	Israel and Germany	3580/3580	N/R	3580 (100%)	Midface lift	3150 (88%)	Overall: 3043 (85%)	5 (0.1%)/0 (0%)	12 (0.3%)/0 (0%)
Waterhouse et al. 2007 [72]	UK	359/359	56.1	327 (91.1%)	Midface lift	337 (93.9%)	N/R	7 (1.9%)/0 (0%)	0 (0%)/0 (0%)
Wong et al. 2021 [73]	Singapoore, Australia	128/128	N/R	N/R	Midface lift	94 (73.4%)	Overall: 102 (79.7%)	2 (1.6%)/0 (0%)	0 (0%)/0 (0%)
Wu et al. 2021 [74]	China	22/22	43.8 (34–53)	22 (100%)	Midface lift	17 (77.3%)	N/R	0 (0%)/0 (0%)	5 (22.7%)/0 (0%)
Wudel et al. 2016 [75]	USA	16/16	62 (40.3–72.7)	14 (87.5%)	Midface lift	N/R	N/R	1 (6.3%)/0 (0%)	0 (0%)/0 (0%)
Yang et al. 2020 [76]	Korea	83/83	63.8 (54–73)	N/R	Midface lift	N/R	N/R	4 (4.8%)/0 (0%)	0 (0%)/0 (0%)
Gualdi et al. 2017 [77]	Italy	55/55	47.1±4.6	50 (90.9%)	Midface lift + Browpexy	N/R	N/R	0 (0%)/0 (0%)	0 (0%)/0 (0%)
Guerrissi 2010 [78]	Argentina	142/142	(35–55)	134 (94.4%)	Midface lift + Browpexy	119 (83.8%)	Blephasropl.: 48 (33.8%)	0 (0%)/0 (0%)	0 (0%)/0 (0%)
Cohen et al. 2011 [79]	USA	21/21	(54–70)	9 (42.9%)	Browpexy	N/R	N/R	0 (0%)/0 (0%)	0 (0%)/0 (0%)
Guyuron et al. 1995 [80]	USA	8/8	51.3 (25–66)	7 (87.5%)	Browpexy	N/R	Blepharopl.: 8 (100%)	0 (0%)/0 (0%)	8 (100%)/0 (0%)
Malata et al. 2009 [81]	UK	30/30	60 (34–76)	23 (76.7%)	Browpexy	N/R	Overall: 12 (40%) Blepharopl.: 8 (26.7%)	0 (0%)/0 (0%)	1 (3.3%)/0 (0%)
Miller et al. 2000 [82]	USA	65/65	N/R	N/R	Browpexy	N/R	N/R	0 (0%)/0 (0%)	0 (0%)/0 (0%)
Shu et al. 2016 [83]	China	496/496	48±8 (38–70)	496 (100%)	Browpexy	N/R	–	0 (0%)/0 (0%)	8 (1.6%)/0 (0%)
Tabatabai et al. 2007 [84]	USA	193/193	N/R	N/R	Browpexy	N/R	N/R	0 (0%)/0 (0%)	1 (0.5%)/1 (0.5%)
Ramirez et al. 1991 [85]	USA	28/28	N/R	N/R	Frontal face lift	N/R	N/R	1 (3.6%)/0 (0%)	0 (0%)/0 (0%)
Ramirez 1992 [86]	USA	34/34	N/R	N/R	Frontal face lift	N/R	N/R	0 (0%)/0 (0%)	1 (2.9%)/0 (0%)
Scheflan et al. 1996 [87]	Israel, Switzerland, France, USA	545/545	N/R	N/R	Frontal face lift	N/R	N/R	47 (8.6%)/11 (2%)	0 (0%)/0 (0%)

*Blepharopl.* Blepharoplasty, *N/R* not reported, *USA* Unites States of America.

The most common type of surgery was midface lift (58 studies), with browpexy in 8 studies and frontal facelift in 4 studies. In 2 publications, all patients underwent a combined midface lift and browpexy approach [77, 78]. A heterogeneity of surgical techniques was described between the different studies, including SMAS plication, SMASectomy, deep-plane rhytidectomy,, composite rhytidectomy and endoscopic procedures, with differences regarding incision location, tensile vectors, and adjunctive procedures. Several studies included patients treated with different surgical techniques. Twenty-one publications reported the number of secondary and tertiary procedures, with three studies including only first-time midface lifts [29, 37, 42] and one including only patients who had previously undergone facelift surgery [25]. Considering additional procedures, in 30 publications at least one patient underwent simultaneous aesthetic surgical procedures, with 7 studies mentioning that all patients had such an intervention [29, 32, 33, 53, 59, 66, 80]. Blepharoplasty was the most commonly reported adjunctive procedure, performed in 24.5 to 100%, depending on the publications.

### Postoperative Neurologic Side Effects

All included studies described postoperative neurological damage, allowing their classification as motor or sensory deficits, as well as permanent or transient side effects. Twenty-five (37.3%) publications reported no neurological side effects due to surgical procedures, transient or permanent, while only seven studies reported permanent neural damage, with a rate ranging from 0.1 to 2%.

**Fig. 2:**
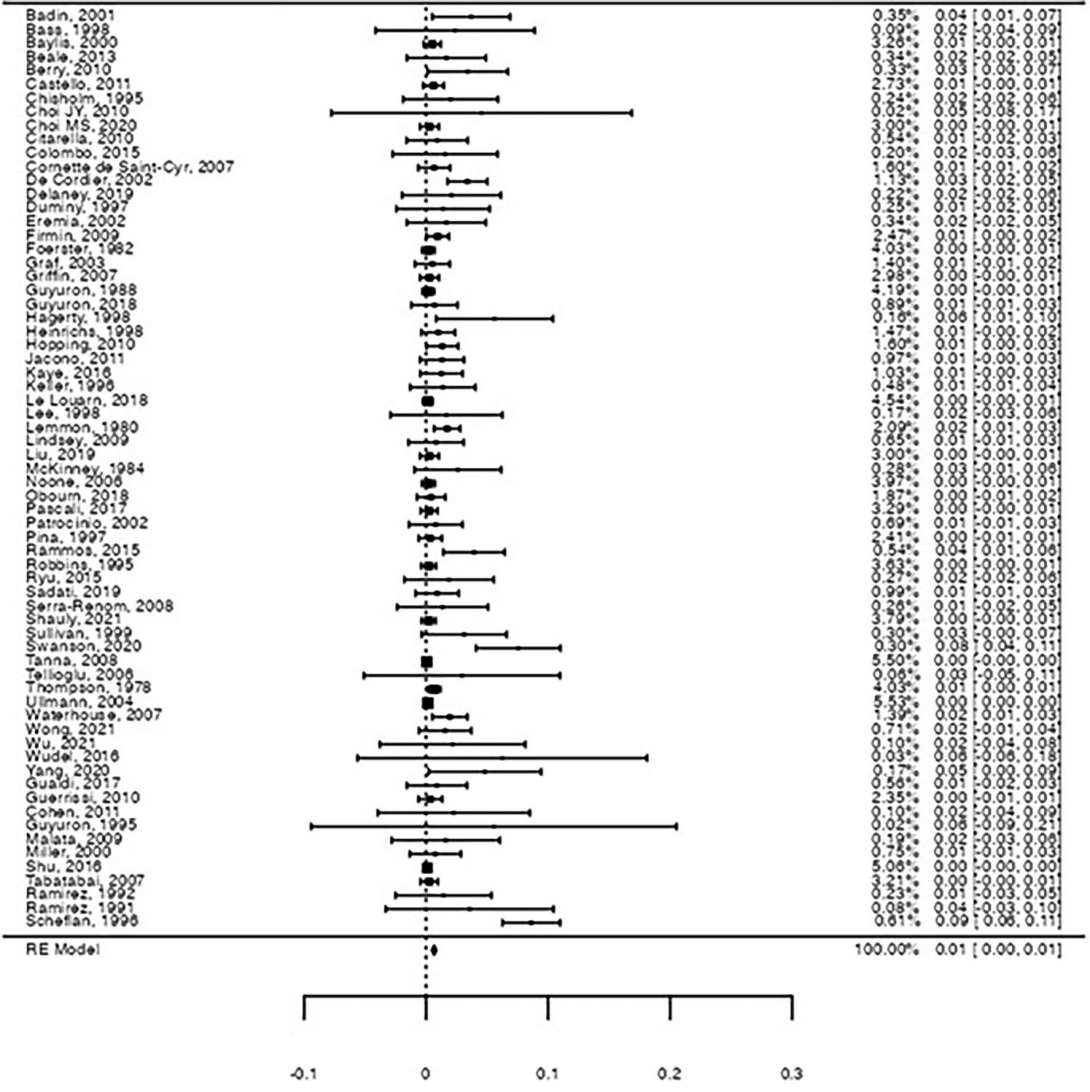
Forest-plot on the raw proportion for overall motor nerve damage. A pooled proportion of 0.66% was observed for motor nerve damage (95%CI: [0.5%; 0.9%], Z = 6.07, *p* < .001), with significant heterogeneity (I^2^ = 59.1%, *Q*(66) = 161, *p* < .001)

Table 2 resumes the results of the pooled rates for motor and sensory nerve damage after the operation, with the inclusion of all 67 studies. The estimated pooled motor nerve damage was 0.66% (Forrest plot in Figure 2, 95% confidence interval [95%CI]: [0.5%; 0.9%], Z = 6.07, *p* < .001), with significantly high heterogeneity between studies (I^2^ = 59.1%, *Q*(66) = 161, *p* < .001). Motor nerve damage was mostly transient and affected different branches of the facial nerve, namely temporal, zygomatic, marginal mandibular, and buccal branches. Some publications reported only facial nerve damage, without specifying the affected branch.

**Table 2:** Metanalysis results for pooled proportions estimates

Endpoint (nerve damage)	Pooled rate (%)	95% confidence interval	Z	*p*-value		Heterogeneity analysis	
					I^2^ (%)	*Q(df)*	*p*-value
Overall motor	0.66	[0.5%; 0.9%]	6.70	<.001	59.1	*Q*(66) = 161	<.001
Overall sensory	0.39	[0.2%; 0.6%]	4.16	<.001	74.1	*Q*(66) = 255	<.001
Permanent motor	0.047	[0.0%; 0.1%]	2.69	.007	0.0	*Q*(66) = 38.8	1.00
Permanent sensory	0.045	[0.0%; 0.1%]	2.63	.009	0.0	*Q*(66) = 28.4	1.00

*df* degrees of freedom

**Fig. 3:**
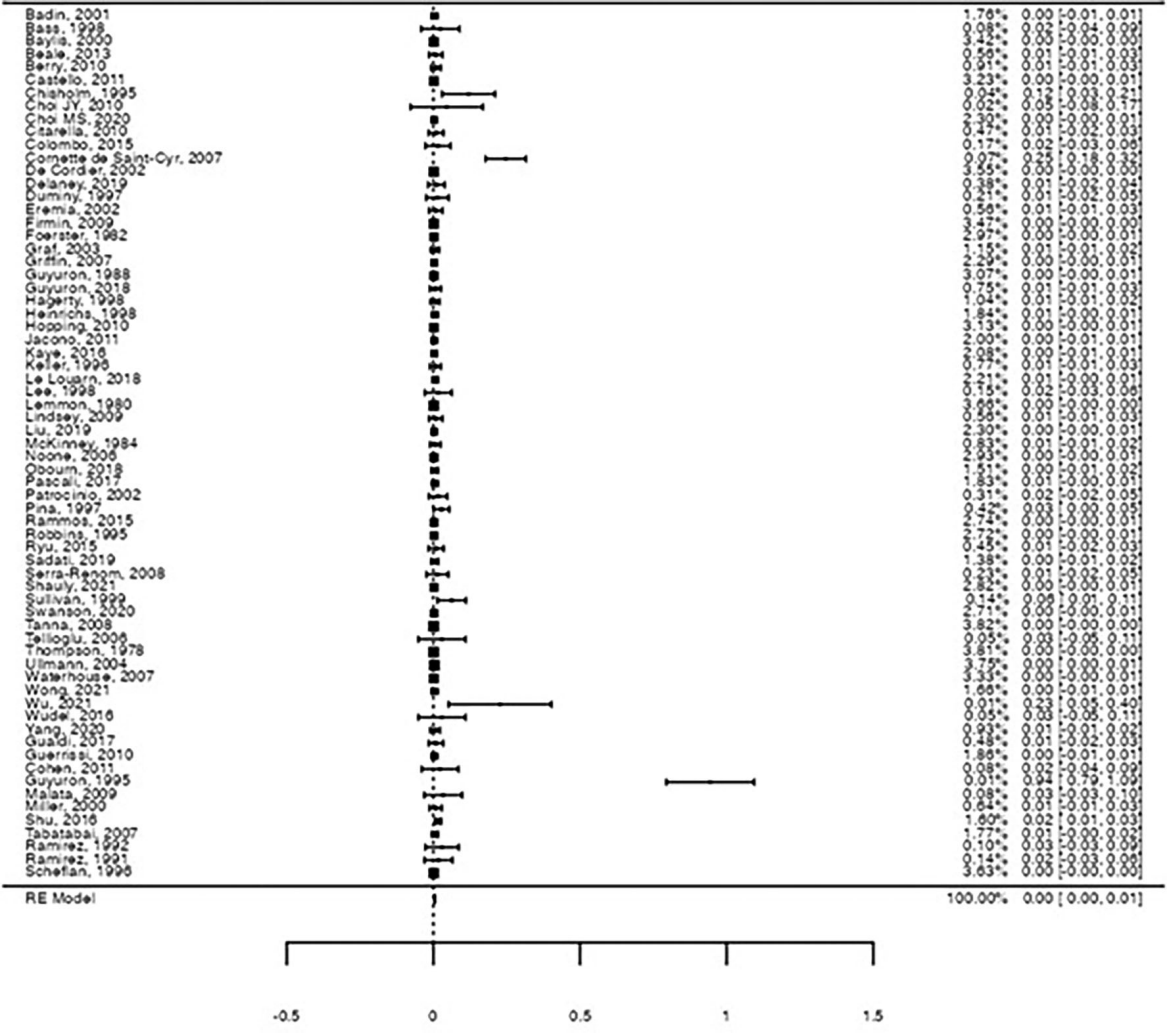
Forest-plot on the raw proportion for overall sensory nerve damage. A pooled proportion of 0.39% was observed for motor nerve damage (95%CI: [0.2%; 0.6%], Z = 4.16, *p* < .001), with significant heterogeneity (I^2^ = 74.1%, *Q*(66) = 255, *p* < .001)

For overall sensory damage, the estimated pooled rate was 0.39% (Forrest plot in Figure 3, 95%CI: [0.2%; 0.6%], Z = 4.16, *p* < .001), also with a significantly high heterogeneity between studies (I^2^ = 74.1%, *Q*(66) = 255, *p* < .001). Postoperative sensitive changes included preauricular, retroauricular, earlobe, inferior eye lid, and midface paresthesia, upper lip, and forehead and scalp dysesthesia.

**Fig. 4:**
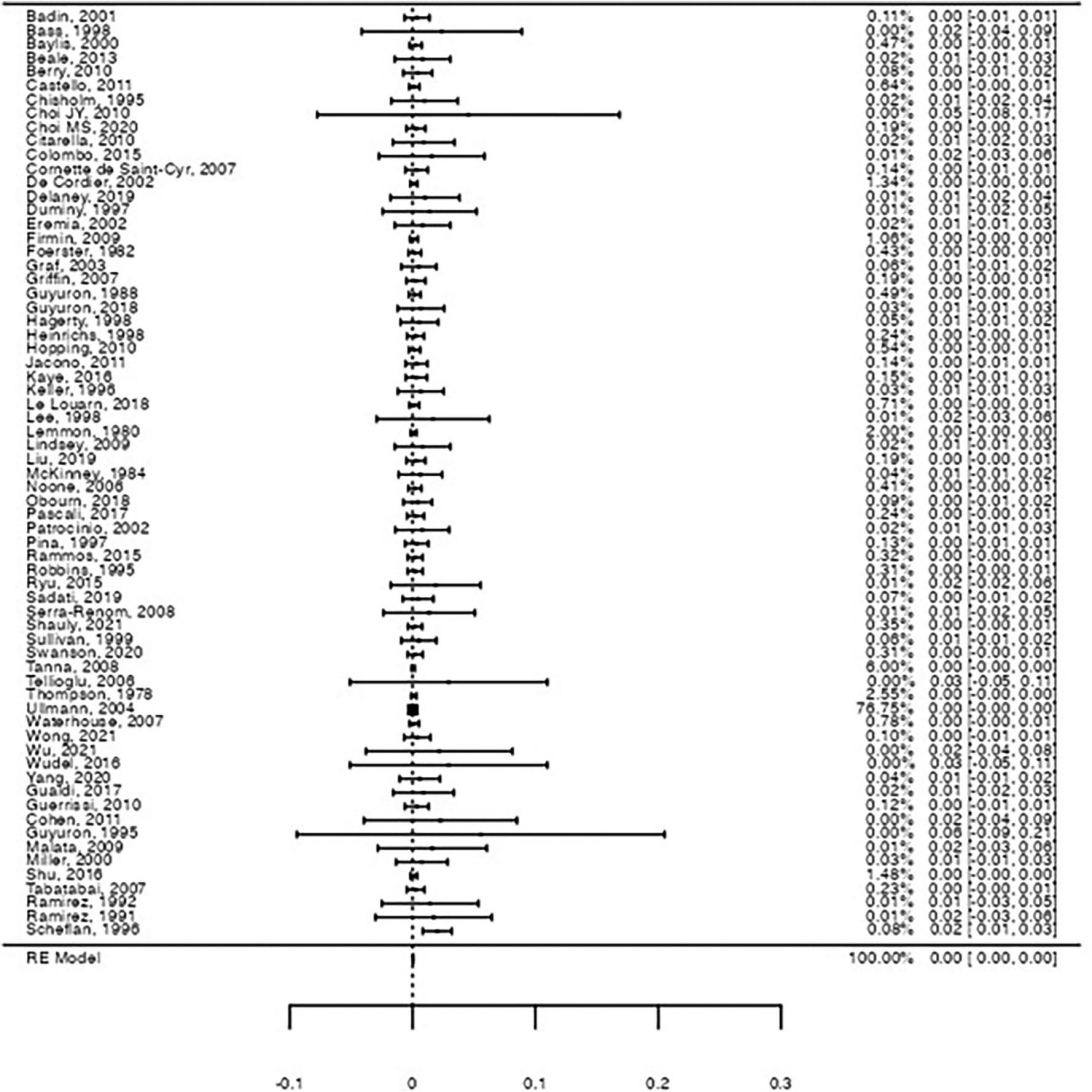
Forest-plot on the raw proportion for permanent motor nerve damage. A pooled proportion of 0.047% was observed for motor nerve damage (95%CI: [0.0%; 0.1%], Z = 2.69, *p* = .007), with no significant heterogeneity (I^2^ = 0%, *Q*(66) = 38.8, *p* = 1.00)

**Fig. 5:**
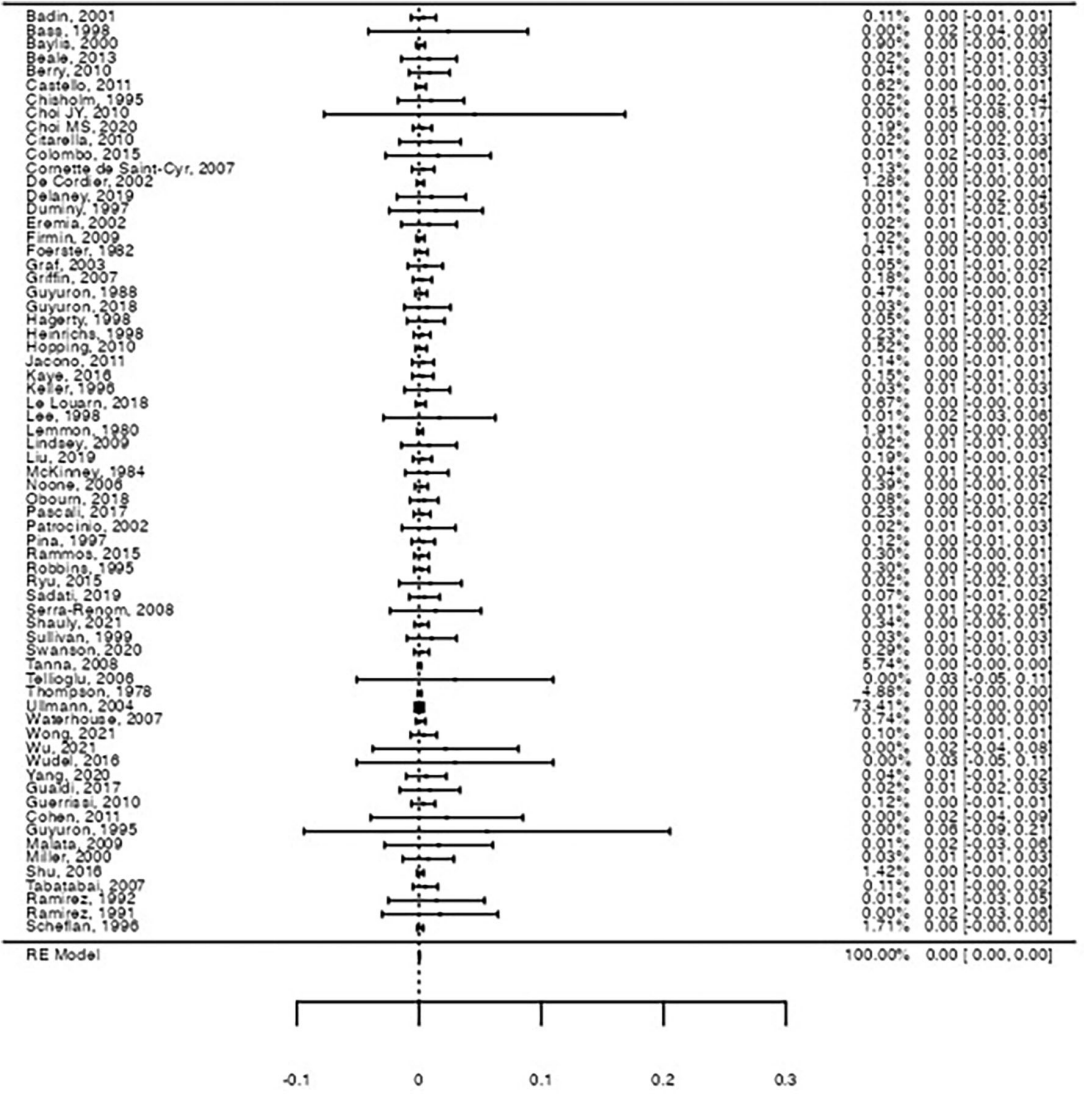
Forest-plot on the raw proportion for permanent sensory nerve damage. A pooled proportion of 0.045% was observed for motor nerve damage (95%CI: [0.0%; 0.1%], Z = 2.63, *p* = .009), with no significant heterogeneity (I^2^ = 0%, *Q*(66) = 28.4, *p* = 1.00)

Considering permanent neuronal damage, the pooled rates were less than 0.1% for both motor and sensory damage. Metanalyses results showed a pooled 0.047% rate for permanent motor damage (Forrest plot in Figure 4, 95%CI: [0.0%; 0.1%], Z = 2.69, p = .007) and of 0.045% for permanent sensory damage (Forrest plot in Figure 5, 95%CI: [0.0%; 0.1%], Z = 2.63, *p* = .009). No study heterogeneity was observed for either model (I^2^ = 0%, *Q*(66) = 38.8, *p* = 1.00 and I^2^ = 0%, *Q*(66) = 28.4, *p* = 1.00, respectively). Permanent damage to the facial nerve for its temporal and marginal mandibular branch was described, with partial recovery on observation, while permanent sensory damage included dysesthesia with chronic pain (sensory region not specified) and paresthesia to the territory of the supraorbital/suprasupratrochlear nerve.

### Risk of Bias

Table 3 presents the analysis of each study for risk of bias. Since most of the studies were retrospective and included patients who underwent a second or third facelift, there could be a confounding bias, as nerve damage could be related to previous interventions. Also, selection bias could be present in two publications, as one of them only included patients also submitted to orthognathic surgery and the other retrospectively selected patients in need of minoxidil to treat temporal alopecia. Publications with a clear retrospective design pointed to a possible risk of information bias, as nerve damage information was based on patient chart review, which implies the possibility of missing data on the measured outcome. No risk of reporting bias was identified since all included studies reported the desired outcome for all patients.

**Table 3:** Risk for bias for the included publications

Authors, year	Confounding bias	Selection bias	Information bias	Reporting bias
Badin et al. 2001 [22]	+	+	–	+
Bass 1998 [23]	–	+	+	+
Baylis et al. 2000 [24]	+	+	–	+
Beale et al. 2013 [25]	–	+	–	+
Berry et al. 2010 [26]	–	+	+	+
Castello et al. 2011 [27]	+	+	–	+
Chisholm et al. 1995 [28]	+	+	+	+
Choi JY et al. 2010 [29]	–	–	–	+
Choi MS et al. 2020 [30]	+	+	–	+
Citarella et al. 2010 [31]	+	+	+	+
Colombo et al. 2015 [32]	+	+	+	+
Cornette de Saint-Cyr et al. 2007 [33]	+	+	+	+
De Cordier et al. 2002 [34]	–	+	–	+
Delaney et al. 2019 [35]	–	+	–	+
Duminy et al. 1997 [36]	+	+	+	+
Eremia et al. 2002 [37]	+	–	–	+
Firmin et al. 2009 [38]	+	+	–	+
Foerster 1982 [6]	+	+	–	+
Graf et al. 2003 [39]	+	+	–	+
Griffin et al. 2007 [40]	+	+	–	+
Guyuron 1988 [41]	+	+	+	+
Guyuron et al. 2018 [42]	–	+	+	+
Hagerty et al. 1998 [43]	–	+	+	+
Heinrichs et al. 1998 [44]	–	+	–	+
Hopping et al. 2010 [45]	+	+	–	+
Jacono et al. 2011 [46]	+	+	–	+
Kaye et al. 2016 [47]	+	+	–	+
Keller et al. 1996 [48]	+	+	–	+
Le Louarn 2018 [49]	+	+	–	+
Lee et al. 1998 [50]	+	+	–	+
Lemmon et al. 1980 [51]	–	+	–	+
Lindsey 2009 [52]	+	+	–	+
Liu et al. 2019 [53]	+	+	+	+
McKinney et al. 1984 [54]	+	+	+	+
Noone 2006 [55]	+	+	+	+
Obourn et al. 2018 [56]	+	+	–	+
Pascali et al. 2017 [57]	+	+	–	+
Patrocínio et al. 2002 [58]	+	+	–	+
Pina 1997 [59]	–	+	–	+
Rammos et al. 2015 [60]	–	+	–	+
Robbins et al. 1995 [61]	+	+	–	+
Ryu et al. 2015 [62]	+	+	+	+
Sadati et al. 2019 [63]	–	+	–	+
Serra-Renom et al. 2008 [64]	+	+	+	+
Shauly et al. 2021 [65]	–	+	+	+
Sullivan et al. 1999 [66]	+	+	–	+
Swanson 2020 [67]	+	+	–	+
Tanna et al. 2008 [68]	–	+	–	+
Tellioglu et al. 2006 [69]	+	+	–	+
Thompson et al. 1978 [70]	+	+	–	+
Ullmann et al. 2004 [71]	–	+	–	+
Waterhouse et al. 2007 [72]	–	+	–	+
Wong et al. 2021 [73]	–	+	+	+
Wu et al. 2021 [74]	–	+	+	+
Wudel et al. 2016 [75]	+	+	–	+
Yang et al. 2020 [76]	+	+	+	+
Gualdi et al. 2017 [77]	+	+	+	+
Guerrissi 2010 [78]	–	+	+	+
Cohen et al. 2011 [79]	+	+	–	+
Guyuron et al. 1995 [80]	+	+	+	+
Malata et al. 2009 [81]	+	+	–	+
Miller et al. 2000 [82]	+	+	+	+
Shu et al. 2016 [83]	+	+	+	+
Tabatabai et al. 2007 [84]	+	+	–	+
Ramirez et al. 1991 [85]	+	+	–	+
Ramirez 1992 [86]	+	+	+	+
Scheflan et al. 1996 [87]	+	+	–	+

**+** no risk of bias, **–** risk for bias.

## Discussion

In recent years, the search for aesthetic surgery has increased, possibly due to improved access to health care and increased peer pressure on aesthetic problems. During surgical consultation, the discussion of the potential side effects of any surgery with the patient is of the greatest importance, as surgical risk must be weighed against the potential surgical benefits, of which the patient must be aware for informed decision-making. Different complications of facelift surgery are described in the literature; We opted to evaluate complications related to nerve injury, as its occurrence, namely permanent nerve damage, has a significant impact on patient quality of life and could be associated with depression [88].

Several retrospective and prospective series describe nerve-related complications of facelift surgeries. However, a wide variety of techniques are described in the literature, with rates of neurologic complication ranging from 0 to 100%, and sample sizes as low as 8 patients. Therefore, a systematic review on this topic was performed, with a metanalysis for the estimation was performed for a better quantify the total and permanent damage.

Due to anatomical considerations, it is predictable that surgical techniques that require a deeper approach to SMAS, such as deep-plane and composite rhytidectomy, may result in a higher rate of nerve damage compared to SMAS plication or SMASectomy procedures. However, the publications included in this review applied a wide variety of surgical techniques, with several studies including patients treated with different approaches, which did not allow separate quantification of nerve lesions per type of surgical procedure. Furthermore, none of the studies compared different surgical techniques with respect to postoperative nerve injury rates. Though, the overall pooled nerve injury in our meta-analysis was less than 1%, and permanent nerve injury less than 0.1%, for both motor and sensory lesions. In fact, the evolution of surgical techniques over time, careful surgical planning and the experience of the plastic surgeon could help preserve postoperative nerve function [89]. A careful selection of the appropriate surgical approach for facelifts could help reduce the rates of nerve injuries [17]. Additionally, the avoidance of four specific anatomical landmarks where nerves become superficial is also related to a lower rate of nerve damage [90].

The first landmark is located inferiorly and laterally to the zygomatic eminence, where the zygomatic nerve branch of the facial nerve becomes superficial. The second is located in the transition from lateral to middle cheek fat, where there is an increased risk of lesion to the buccal branch of the facial nerve. The third location is around the inferior masseteric border, where the marginal mandibular branch leaves the parotid tail within the sub-SMAS fat. The best way to avoid any damage in this region is to keep the dissections of the platysma-SMAS superficial. The marginal branch is also at risk in the fourth danger zone, at the cheek-chin junction. Along these areas, it is fundamental to also take into account the cervical and frontal branches. The cervical branch, due to its superficial location and attachment to the platysma, is at high risk for damage, limited by approaching the SMAS with lateral incisions along the platysma and using the mandibular angle as a visual cue. The frontal branch is considered to be one of the most vulnerable, particularly in rhytidectomies in deeper layers of the fascia; nerve damage can be minored with an approach starting from the inferior level of the temporoparietal fascia. Intraoperative protocols emphasize the preservation of nerves, primarily employing bipolar cautery to mitigate thermal injury. Additionally, superficial postauricular dissection is utilized to safeguard the great auricular nerve, while staged hemostasis through a "second look" approach is adopted to manage rebound bleeding [2, 17, 90].

Although rare, nerve lesions must be treated appropriately [91]. The administration of corticosteroid within 24 hours of injury reduces inflammation and improves the likelihood of recovery. Permanent sensory damage usually does not affect quality of life, since it is mainly residual [92]. Motor nerve injuries are treated by surgical or non-surgical interventions. Adequate facial symmetry can be achieved through subsequent surgical aesthetic interventions or chemodenervation with botulinic toxin [91–93]. The immediate intraoperative recognition of the injury allows primary neurorrhaphy (direct nerve suturing) or nerve grafts (e.g. sural or great auricular nerve) to bridge the gaps, achieving partial to complete functional recovery. For delayed presentations, nerve transfers (e.g., hypoglossal-facial) can restore function [87, 94]. Physical therapy can also be used, although a systematic review was unable to establish its benefit in iatrogenic facial paralysis [95].

To achieve satisfactory results, facelift surgery is generally performed at the same time as other aesthetic surgeries. In fact, several publications report adjunctive procedures such as blepharoplasty, rhinoplasty, and browpexy in up to 100% of cases. However, the need to handle other surgical planes at the same time might influence the postoperative complication rate [96]. Therefore, including patients who undergo simultaneous procedures can limit precision in establishing a clear cause effect between facelift surgery and transient/permanent nerve damage.

Few patients report unsatisfactory results after facelift surgery, in which cases resurgery or secondary (or additional) facelift procedures are needed [63]. In this review, at least 21 publications included patients undergoing non-primary facelifts. Although no description of nerve damage from the preceding surgery was reported in these studies, previous surgical manipulation could influence the rate of nerve-related postoperative complications, which can be considered a limitation in determining the true rate of neurological complications of facelift surgery.

In this systematic review, many retrospective studies were included, which might account for some missing values in reporting postoperative events. Nevertheless, postoperative neural damage is clearly described as one possible complication of facelift surgery, and the possibility of omission on patient charts is unlikely due to the high impact on the patient’s daily routine.

## Conclusion

Nerve damage is one of the most feared complications of facelift procedures, as facial motor and sensitive changes have a significant impact on patient’s quality of life. Plastic surgeons are increasingly aware of this serious complication, with the use of surgical techniques that achieve better and longer-lasting results and minimize nerve-related injuries. In fact, this systematic review with metanalysis establishes that nerve damage constitutes a rare postoperative complication of facelift surgery, with an overall pooled rate of less than 1% and a permanent nerve injury rate of less than 0.1%, for motor and sensory nerve lesions. For motor injuries, acute transections are best treated with primary neurorrhaphy within 72 hours to optimize recovery by ensuring proper axonal regrowth. Additionally, botulinum toxin effectively addresses muscle imbalances and improves facial symmetry in cases of persistent deficits. Most sensory lesions resolve spontaneously within a year, with permanent deficits being rare and generally not affecting quality of life.

Surgical techniques are constantly evolving with the aim of optimizing both aesthetic and functional results. This accounts for several variations of facelift surgical procedures over the years, limiting conclusions on the true rate of complications for each technique; also, several reports on iatrogenic nerve injury in this scenario are retrospective. More prospective studies are needed to compare different surgical techniques with respect to the rates of neurologic side effects, in order to identify the most appropriate technique to achieve an optimized result.

## Declarations

### Research Involving Human and Animal Participants

This article does not contain any studies with human participants or animals performed by any of the authors.
